# Metabolic Profile of Leaves and Pulp of *Passiflora caerulea* L. (Bulgaria) and Their Biological Activities

**DOI:** 10.3390/plants13131731

**Published:** 2024-06-22

**Authors:** Anelia Gerasimova, Krastena Nikolova, Nadezhda Petkova, Ivan Ivanov, Ivayla Dincheva, Yulian Tumbarski, Velichka Yanakieva, Mina Todorova, Galia Gentscheva, Anna Gavrilova, Ina Yotkovska, Stoyanka Nikolova, Pavlo Slavov, Nikolay Harbaliev

**Affiliations:** 1 Department of Chemistry, Faculty of Pharmacy, Medical University—Varna, 9000 Varna, Bulgaria; anelia.gerasimova@mu-varna.bg; 2 Department of Physics and Biophysics, Faculty of Pharmacy, Medical University—Varna, 9000 Varna, Bulgaria; 3 Department of Organic Chemistry and Inorganic Chemistry, University of Food Technologies, 4002 Plovdiv, Bulgaria; nadezhda_petkova@uft-plovdiv.bg (N.P.); ivanov_ivan.1979@yahoo.com (I.I.); 4 Department of Agrobiotechnologies, Agrobioinstitute, Agricultural Academy, 1164 Sofia, Bulgaria; vadincheva@yahoo.com; 5 Department of Microbiology, University of Food Technologies, 4002 Plovdiv, Bulgaria; tumbarski@abv.bg (Y.T.); yanakieva_vili@abv.bg (V.Y.); 6 Department of Organic Chemistry, Faculty of Chemistry, University of Plovdiv, 4000 Plovdiv, Bulgaria; minatodorova@uni-plovdiv.bg (M.T.); tanya@uni-plovdiv.bg (S.N.); 7 Department of Chemistry and Biochemistry, Medical University—Pleven, 5800 Pleven, Bulgaria; ina.iotkovska@mu-pleven.bg; 8 Department of Pharmaceutical Chemistry and Pharmacognosy, Medical University—Pleven, 5800 Pleven, Bulgaria; anna.gavrilova@mu-pleven.bg; 9 Faculty of Medicine, Medical University—Varna, 9000 Varna, Bulgaria; pavloslavov@gmail.com (P.S.); harbaliev.n@gmail.com (N.H.)

**Keywords:** *Passiflora caerulea* L., antimicrobial activity, antioxidant activity, chemical composition, anti-inflammatory activity, total phenolic content

## Abstract

At present, there are no data in the scientific literature on studies aimed at characterizing *Passiflora caerulea* L. growing in Bulgaria. The present study aimed to investigate the metabolic profile and elemental composition of the leaves and pulp of this *Passiflora*, as well as to evaluate the antioxidant, antimicrobial and anti-inflammatory activities of its leaf and pulp extracts. The results showed that the pulp predominantly contained the essential amino acid histidine (7.81 mg g^−1^), while it was absent in the leaves, with the highest concentration being tryptophan (8.30 mg g^−1^). Of the fatty acids, palmitoleic acid predominated both in the pulp and in the leaves. A major sterol component was β-sitosterol. Fructose (7.50%) was the predominant sugar in the pulp, while for the leaves, it was glucose—1.51%. Seven elements were identified: sodium, potassium, iron, magnesium, manganese, copper and zinc. The highest concentrations of K and Mg were in the pulp (23,946 mg kg^−1^ and 1890 mg kg^−1^) and leaves (36,179 mg kg^−1^ and 5064 mg kg^−1^). According to the DPPH, FRAP and CUPRAC methods, the highest values for antioxidant activity were found in 70% ethanolic extracts of the leaves, while for the ABTS method, the highest value was found in 50% ethanolic extracts. In the pulp, for all four methods, the highest values were determined at 50% ethanolic extracts. Regarding the antibacterial activity, the 50% ethanolic leaf extracts were more effective against the Gram-positive bacteria. At the same time, the 70% ethanolic leaf extract was more effective against Gram-negative bacteria such as *Salmonella enteritidis* ATCC 13076. The leaf extracts exhibited higher anti-inflammatory activity than the extracts prepared from the pulp. The obtained results revealed that *P. caerulea* is a plant that can be successfully applied as an active ingredient in various nutritional supplements or cosmetic products.

## 1. Introduction

The genus *Passiflora* (*Passifloraceae)* includes about 500 species, which are mainly distributed in the tropical and subtropical regions of South America but are also found in North America, the West Indies, the Galapagos Islands, Africa, Australia, the Philippines, Asia and many islands in the Pacific Ocean [1,2]. Some of the species are widely used in traditional South American medicine, generally to treat nervousness, insomnia, anxiety and related disorders [3,4]. Since the XVII century, a few Passiflora species have been used in Europe, as well, mainly due to their soothing properties [4,5].

*Passiflora caerulea* L. (blue passion flower) is native to the temperate regions of the South American countries of Argentina, Brazil, Bolivia, Chile, Paraguay and Uruguay. The species is a woody vine that can reach up to 25 m in height if other support is available. The leaves are alternate, palmate, usually have five lobes and are up to 10 cm in length. Each leaf has double tendrils 5–10 cm long at its base, which help the plant to climb up supporting vegetation. The flower is complex, about 10 cm in diameter, with the five sepals and petals similar in appearance, whitish in color and surmounted by a corona of blue or violet filaments, with five greenish-yellow stamens and three purple stigmas (7–12). The fruit is an oval orange-yellow berry with numerous seeds and is 6 cm long and 4 cm in diameter. It is edible to humans when ripe but tends to have an undesirable flavor [2].

*P. caerulea* is currently cultivated worldwide as an ornamental plant, but it possesses both nutritional value and medicinal properties [2,6]. The most popular traditional uses of blue passion flower, mainly in Latin America, are as a sedative, anxiolytic, antispasmodic, antihypertensive and diuretic agent, a remedy for gastrointestinal disorders, vermifuge and an antimicrobial agent against catarrh and pneumonia [5,6,7,8,9,10]. Decoction of *P. caerulea* is used as a mild antimicrobial agent in the folk medicine of Argentina [11]. Additionally, some recent studies have revealed the anti-inflammatory, anti-diarrheal, spasmolytic, anticonvulsant, analgesic and antioxidant effects of extracts from leaves or entre aerial parts [6,12,13]. The fruit extracts show anticonvulsant activity, improvement of cognitive function, oxidative damage reduction and cholinergic neurotransmission activation [14].

The different parts of the *P. caerulea* have rich phytochemical content. The leaves and aerial parts, in general, contain chrysin, luteolin, quercetin, apigenin, myricetin and C-glycosylated flavonoids (orientin, isoorientin, vitexin, isovitexin, etc.), alkaloids, saponins, triterpenoids, cyanogenic acids, phenolics, etc. [2,4,9,15,16]. The fruit contains epigallocatechin gallate, ginsenoside, naringenin, chrysoeriol, luteolin, apigenin, carbohydrates mainly composed of dietary fibers, pectin, carotenoids (lycopene, α- and β-carotene, β-cryptoxanthin, zeaxantin, etc.), minerals, etc. [14,17,18]. Some authors assert that the environmental conditions of growth significantly affect the metabolic profile of *P. caerulea* [1,2].

In Bulgaria, *P. caerulea* is grown locally as an ornamental plant across the Black Sea coast, which is characterized by a continental–Mediterranean climate [19]. However, in recent years, it has appeared more and more often in some inner parts of the country with transitional–continental and temperate–continental climates, such as the towns of Plovdiv, Veliko Tarnovo, Sliven, Vratsa, Pleven, etc. Although the species distribution in the country can be estimated as limited at the moment, the currently evolving worldwide climate change could become a factor for its spontaneous expansion out of the loci of initial introduction. The reference in this regard shows that some other *Passiflora* species in recent years have been established as being invasive in specific regions, such as *P. edulis Sims* in the Galapagos archipelago [20], *P. alata Curtis* in Southern Brazil [21], *P. tripartita var mollissima* (Kunth) Holm-Niels. and P.M.Jørg. in New Zealand [22,23], *P. foetida* L. in Australia and several countries of Africa and Southeast Asia [24,25] and *P. tarminiana* Coppens and V.E. Barney in the Hawaiian archipelago [26]. On the other hand, there are some well-known invasive species alien to the Bulgarian flora that originated from areas with temperate climates on other continents, such as *Acer negundo* L., *Ailanthus altissima* (Mill.) Swingle, *Amorpha fruticosa* L., *Fallopia x bohemica* Chrtek and Chrtková, *Xanthium orientale* L. and *X. spinosum* L. Now, some of them are distributed abundantly in the territory of the whole country [27]. A reasonable and economically sound way to help control these species’ distribution is to find ways for their utilization as a source of biologically active ingredients, as they provide an accessible and inexpensive resource [28,29].

The aim of the presented study is to elucidate the metabolic profile, elemental composition and related biological activities (antioxidant, antimicrobial and anti-inflammatory) of various extracts from leaves and pulp of *Passiflora caerulea* L. sourced from plants that were introduced in Bulgaria as ornamental.

The current study is the first of its kind on *P. caerulea* in Bulgaria. Therefore, the obtained results can increase the knowledge about this species and discover opportunities for its utilization in nutritional supplements.

## 2. Material and Methods

### 2.1. Plant Collection and Identification

**Figure 1:**
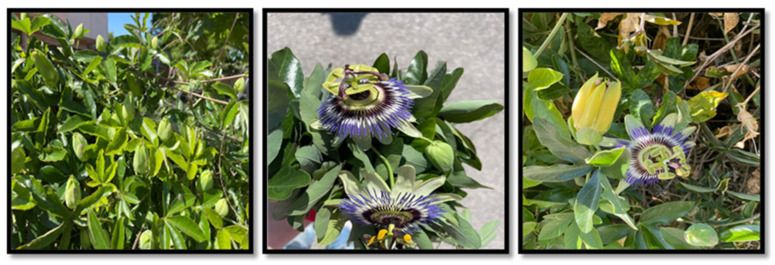
Appearance of *Passiflora caerulea* L.—leaves and flower.

Мature fruit of *Passiflora caerulea* L. was collected in September 2022 in the Trakata area (43.22237 N; 27.97573 E) near Varna, Bulgaria. The plant material (Figure 1) for the presented research was identified by Asst. Prof. Anna Gavrilova, PhD, from the Faculty of Pharmacy, Medical University—Pleven. The voucher specimen (CO 1423) was deposited in the Herbarium of the Institute of Biodiversity and Ecosystem Research (IBER) at the Bulgarian Academy of Sciences (SOM).

### 2.2. Drying

The leaf and fruit pulps of *P. caerulea* L. were dried at 40 °C in a dryer to a constant weight. After that, they were homogenized and ground, packed in cloth bags, and stored in darkness at 20 °C.

### 2.3. Microorganisms

Sixteen microorganisms, including five Gram-positive bacteria (*Bacillus subtilis* ATCC 6633, *Bacillus cereus* NCTC 11145, *Staphylococcus aureus* ATCC 25923, *Listeria monocytogenes* NBIMCC 8632, *Enterococcus faecalis* ATCC 29212), five Gram-negative bacteria (*Salmonella enteritidis* ATCC 13076, *Klebsiella pneumoniae* ATCC 13883, *Escherichia coli* ATCC 25922, *Proteus vulgaris* ATCC 6380, *Pseudomonas aeruginosa* ATCC 9027), two yeasts (*Candida albicans* NBIMCC 74, *Saccharomyces cerevisiae* ATCC 9763) and four fungi (*Aspergillus niger* ATCC 1015, *Aspergillus flavus*, *Penicillium chrysogenum*, *Fusarium moniliforme* ATCC 38932) from the collection of the Department of Microbiology at the University of Food Technologies, Plovdiv, Bulgaria, were selected for the antimicrobial activity test.

*B. subtilis* and *B. cereus* were cultured on Luria–Bertani agar with glucose (LBG agar) at 30 °C for 24 h, while *S. aureus*, *L. monocytogenes*, *E. faecalis*, *S. enteritidis*, *K. pneumoniae*, *E. coli*, *P. vulgaris* and *P. aeruginosa* were cultured on LBG agar at 37 °C for 24 h. *C. albicans* was cultured on Malt extract agar (MEA) at 37 °C, while *S. cerevisiae* was cultured on MEA at 30 °C for 24 h. The fungi *A. niger*, *A. flavus*, *P. chrysogenum* and *F. moniliforme* were grown on MEA at 30 °C for 7 days or until sporulation. 

### 2.4. Culture Media

Luria–Bertani agar with glucose (LBG agar) (Laboratorios Conda S.A., Madrid, Spain) was used for the cultivation of test bacteria. A total of 50 g of LBG agar base was dissolved in 1 L of deionized water (pH 7.5 ± 0.2). Malt extract agar (MEA) MEA (Scharlab S.L., Barcelona, Spain) was used for cultivation of test yeasts and fungi. A quantity of 48 g of MEA base was dissolved in 1 L of deionized water (pH 5.6 ± 0.2). The culture media were prepared according to the manufacturers’ instructions and autoclaved at 121 °C for 20 min before use.

### 2.5. Methods Used

#### 2.5.1. GC-MS

**Figure 2:**
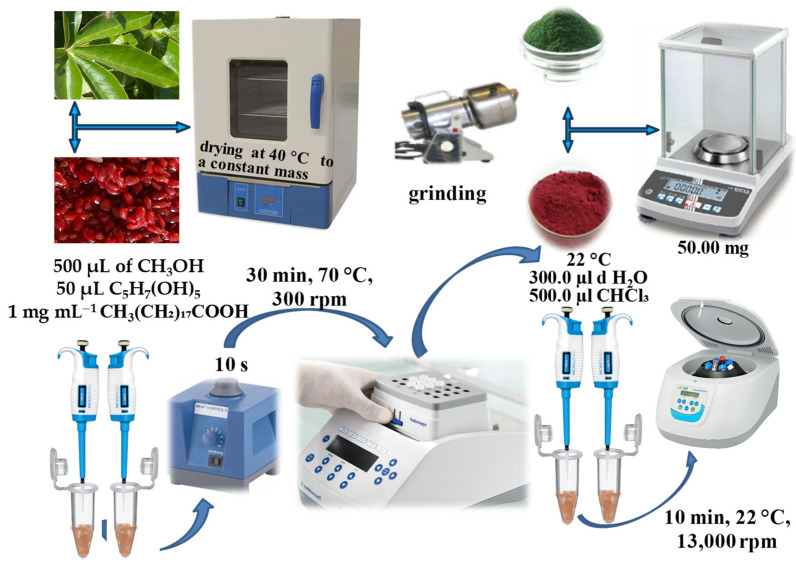
Sample preparation.

A total of 50 mg of dried leaves/fruit pulp was mixed with 500 µL solution of methanol–water (70:25), and 50 µL of internal standard ribitol and nonadecanoic acid, each with concentrations of 1.0 mg mL^−1^, was added. A vortex (IKA - Werke Gmbh & Co. KG, Staufen, Germany) was used to homogenize the mixture for 10 s and was incubated for 30 min at 70 °C and 300 rpm. After cooling to room temperature, 300 µL of distilled water and 500 µL of chloroform were added. The resulting sample was centrifuged at 22 °C and 13,000 rpm for 10 min. Figure 2 illustrates the described process, including the initial sample preparation.

##### Polar Phase

A total of 300.0 µL was carefully pipetted from the supernatant and evaporated under vacuum to dryness. Then, 100.0 µL of 20 mg mL^−1^ solution of methoxyamine was added to this residue. Mixture was incubated under the following conditions: 60 min/70 °C/300 rpm. Then, 50.0 µL of BSTFA sialylation reagent was added, and the mixture was incubated at 70 °C for 40 min, cooled to room temperature and mixed with 300 µL of chloroform. A volume of 1.0 µL was taken from the resulting solution and introduced into the chromatographic system.

##### Nonpolar Phase

A total of 300.0 µL from the supernatant was carefully pipetted and evaporated under vacuum to dryness. Then, 1.0 mL of a 2% sulfuric acid solution in methanol was added to this residue. The mixture was incubated for 60 min at 90 °C/300 rpm. After cooling to room temperature, the sample was extracted three times with hexane (3 × 300 µL) and dried under vacuum at 30 °C. Then, 50.0 µL of pyridine and 50.0 µL of BSTFA were added, and the mixture was incubated at 70 °C for 40 min. After cooling to room temperature, 300 µL chloroform was added. A total of 1.0 µL was taken from the resulting solution and introduced into the chromatographic system.

The solutions were injected into a system consisting of a 7890 A gas chromatograph (Agilent Technologies Inc., Santa Clara, CA, USA) and a 5975C mass spectral detector (Agilent Technologies). An HP-5ms column was used with the following parameters: length of 30 m, diameter of 0.32 mm and film coating thickness of 0.25 µm, with the following temperature program: initial temperature of 60 °C, hold 0 min, rise to 300 °C at 5 °C min^−1^, hold 10 min; injector and detector temperatures of 250 °C; carrier gas helium with a flow rate of 1.0 mL min^−1^; mass detector scan range of *m*/*z* = 50–550; injected sample volume of 1 µL in split mode (split 1:10).

#### 2.5.2. HPLC-RID of Sugars

The extraction procedure was performed in a solid-to-liquid ratio of 1:5 (*w*/*v*) with distilled water in an ultrasonic bath (Siel, Gabrovo, Bulgaria) with frequency of 36 kHz and 300 W power at 65 °C in triplicate. The samples were filtered, and the final volume of leaf and fruit extracts was checked. The analysis of mono- and disaccharide in aqueous extracts was performed on an HPLC instrument, Elite Chrome Hitachi, with mobile-phase distilled H_2_O with a flow rate of 1.0 mL min^−1^ [30]. HPLC-RID method for determination of inulin and fructooligosacharides. ASN, 1, (99–107). Sweetness index (SI) and total sweetness index (TSI) were calculated after HPLC analysis of individual sugars for determination of the fruit sweetness perception [31]. Sweetness Index (SI) was calculated as follows:

SI = (1.00 × [glucose]) + (2.30 × [fructose]) + (1.35 × [sucrose]) (1)

Total sweetness index TSI is calculated according to the equation

TSI = (1.00 × [sucrose]) + (0.76 × [glucose]) + (1.50 × [fructose]) (2)

#### 2.5.3. Determination of Moisture, pH, Ash Contents and Titratable Acidity

Moisture content and ash content (%) were determined according to AOAC [32]. pH was measured using a pH meter 7110 WTW (Weilheim, Germany). Titratable acidity (TA) was measured potentiometrically by titration with 0.1 mol L^−1^ NaOH to the pH value of 8.1 [33].

#### 2.5.4. Determination of Chlorophyll and Pigments

Ethanol extracts were prepared in a ratio of 1:50 (sample/solvent) to determine the content of chlorophyll *a* (C*_a_*), chlorophyll *b* (C_b_) and total carotenoids (C*_x+b_*). For this purpose, 96% pure ethanol (Merck, Germany) was used. For the determination of the content of pigments, absorbance at wavelengths 662 nm, 645 nm and 470 nm was measured, and Equations (3)–(5) reported by [33] were used.

(3) $$C_{a},\mu g\;{mL}^{-1}=13.95\times A_{665}-6.88\times A_{649}$$

(4) $$C_{b},\mu g\;{mL}^{-1}=24.96\times A_{649}-7.32\times A_{665}$$

(5) $$C_{x+b},\mu g\;{mL}^{-1}=\left(1000\times A_{470}-2.05\times C_{a}-114.8\times C_{b}\right)/245$$

#### 2.5.5. Antioxidant Activity

DPPH radical-scavenging ability. The methanol extract (0.15 mL) was mixed with 2.85 mL of fresh 0.1 mM methanol solution of DPPH and analyzed as previously described [34]. 

ABTS radical scavenging ability. Freshly prepared ABTS solution (2.85 mL) was mixed with 0.15 mL of methanol extracts. After 15 min at 37 °C in darkness, the absorbance was measured at 734 nm [34].

FRAP assay was performed as FRAP reagent (3.0 mL) was mixed with 0.1 mL extract, and after 10 min at 37 °С in darkness, the absorbance was recorded at 593 nm [34]. 

CUPRAC assay. The methanol extract (0.1 mL) was mixed with 1 mL of 10 mM CuCl_2_, 1 mL of 7.5 mM methanol solution of Neocuproine, 1 mL of 0.1 M ammonium acetate buffer and 1 mL of distilled H_2_O. After 20 min at 50 °С, the absorbance was measured at 450 nm. 

All the results from antioxidant activity were performed in triplicates and shown as mM Trolox equivalents (mM TE) on g of dry weight (dw) [34].

#### 2.5.6. Total Phenolic Contents (TPC) and Total Flavonoid Contents (TFC)

The TPC was analyzed following the method of Kujala et al. [35] with some modifications. Each extract (0.1 mL) was mixed with 0.5 mL of Folin–Ciocalteu reagent and 0.4 mL of 7.5% Na_2_CO_3_. The mixture was vortexed and left for 5 min at 50 °C. After incubation, the absorbance was measured at 765 nm. The TPC was expressed as mg gallic acid equivalents (GAEs) per g dw.

The total flavonoid content was evaluated according to the method described by Kivrak et al. [36]. An aliquot of 0.5 mL of the sample was added to 0.1 mL of 10% Al(NO_3_)_3_, 0.1 mL of 1 M CH_3_COOK and 3.8 mL of ethanol. After incubation at room temperature for 40 min, the absorbance was measured at 415 nm. Quercetin (QE) was used as a standard, and the results are expressed as mg of quercetin equivalents (QE)/g dw.

#### 2.5.7. Inhibition of Albumin Denaturation

The anti-inflammatory activity was determined by anti-denaturation assay, according to Milusheva et al. [37,38]. The reaction mixture contained 0.5 mL of a 5% aqueous solution of human albumin (Albunorm 20, Octapharma (IP) SPRL, 1070 Anderlecht, Belgium) and 0.2 mL of the tested Passiflora extracts, which were diluted in DMSO at a concentration of 10 mg mL^−1^. The samples were incubated at 37 °C for 15 min. Each tube was filled with 2.5 mL of phosphate-buffered saline (pH 6.3), heated for 30 min to 80 °C and then cooled for 5 min. The turbidity of the samples was measured spectrophotometrically at 660 nm (Cary 60 UV-Vis, Agilent Technologies, Santa Clara, CA 95051, USA). A mixture of 2.5 mL of buffer and 0.2 mL of DMSO was used for the blank, while the product control contained 0.5 mL of serum albumin and 2.5 mL of buffer.

The percent inhibition of protein denaturation was calculated according to Formula (6):

(6) $$Percentage\;of\;inhibition\;denaturation=\frac{\left(Absorbance\;control-Absorbance\;sample\right)}{Absorbance\;control}$$

The control represents 100% protein denaturation. Commercially available anti-inflammatory drugs, diclofenac sodium and acetylsalicylic acid, were used for comparison. Their anti-inflammatory effect was determined as a percentage of inhibition of albumin denaturation, following the same protocol as for the novel compounds.

#### 2.5.8. Antimicrobial Activity Assay

Extracts were obtained after steeping 1 g of ground dry leaves or pulp of *Passiflora caerulea* L. with 10 mL of methanol, 50% or 70% ethanol (Merck, Darmstadt, Germany). The samples were vortexed (Vortex, Ika, model Vortex 3, Germany) for 30 s and left in the dark at room temperature for 72 h. The resulting extracts were filtered through filter paper and stored at 4 °C. Before use, methanol and ethanol were evaporated under vacuum, after which DMSO was added.

The antimicrobial activity of the extracts was determined by the agar well diffusion method [39]. The inocula were prepared by homogenization of a small amount of bacterial/yeast biomass in 5 mL of sterile 0.5% NaCl. The fungal inocula were prepared by the addition of 5 mL of sterile 0.5% NaCl directly into the cultivation tubes. After stirring by vortex V-1 plus (Biosan, Riga, Latvia), the fungal inocula were filtered and replaced in new tubes before use. The number of viable cells and fungal spores was determined using a bacterial counting chamber Thoma (Poly-Optik, GmbH, Bad Blankenburg, Germany), and then suspensions containing approximately 1 × 10^8^ cfu mL^−1^ for bacterial/yeast cells and 1 × 10^5^ cfu mL^−1^ for fungal spores were prepared. Next, the inocula were distributed in preliminarily melted and tempered at 45–48 °C LBG/MEA media and transferred in quantity of 18 mL in sterile Petri plates (d = 90 mm) (CORNING Gosselin SAS, Borre, France). After allowing the inoculated agar media to harden at room temperature, six wells (d = 6 mm) per Petri plate were bored, and duplicates of 60 μL of the extracts were pipetted into the agar wells. The plates were incubated at identical conditions.

The antibiotics Ampicillin and Penicillin (against bacteria) and Nystatin and Fluconazole (against yeast and fungi) in concentrations of 10 mg mL^−1^ were used as controls.

The antimicrobial activity was determined by measuring the diameter of the inhibition zones (IZ) around the wells on the 24th and 48th hour of incubation. Test microorganisms with IZ of 18 mm or more were considered sensitive; moderately sensitive were those in which the IZ were from 12 to 18 mm; resistant were those in which the IZ were up to 12 mm or completely missing.

#### 2.5.9. Determination of Elements

A dry sample of about 0.5 g was decomposed according to the procedure described by Gentscheva et al. [40], and the final sample volume was 50 mL. ICP-OES iCAP 7000 SERIES (Thermo Fisher Scientific, Waltham, MA, USA) was used to determine the concentrations of seven elements in leaves and pulp. After appropriate dilutions, instrument calibration was performed using multi-element ICP standard solution IV (Supelco, Merck, Darmstadt, Germany).

### 2.6. Statistical Analysis

Three parallel measurements were made for each of the studied parameters. Results were presented as mean ± standard deviation (SD). Duncan’s test for multiple comparisons was performed to determine statistically significant differences between extracts for anti-inflammatory and antioxidant activity. Statistical analysis was performed with SPSS v. 24 (IBM Analytics, USA). The results have a significance level of *p* < 0.05.

## 3. Results

Table 1 presents the amount of sugar, pigments such as carotenoids and chlorophyll *a* and *b*, and others.

The content of fatty acids, amino acids, phenolic acids, sterols, etc. in the leaves and pulp of *P. caerulea* is presented in Table 2 and Table 3.

The antioxidant activity, total phenolic content and total flavonoid content of different *P. caerulea* extracts are presented in Table 4. 

The results presented in Table 5 showed that the methanolic and 50% and 70% ethanolic extracts of leaves and pulp of *P. caerulea* demonstrated limited antimicrobial activity (inhibition zones < 12 mm) compared to the antibiotics used as controls.

The leaf extracts did not inhibit the Gram-positive bacteria *S. aureus* ATCC 25923, *L. monocytogenes* NBIMCC 8632, nor the Gram-negative *K. pneumoniae* ATCC 13883 or *P. vulgaris* ATCC 6380. Similar results were obtained for the inhibitory activity of pulp extracts, which did not inhibit either the Gram-positive bacterium *E. faecalis* ATCC 29212. Only the pulp extracts exhibited antifungal activity against *P. chrysogenum* and *F. moniliforme* ATCC 38932.

**Figure 3:**
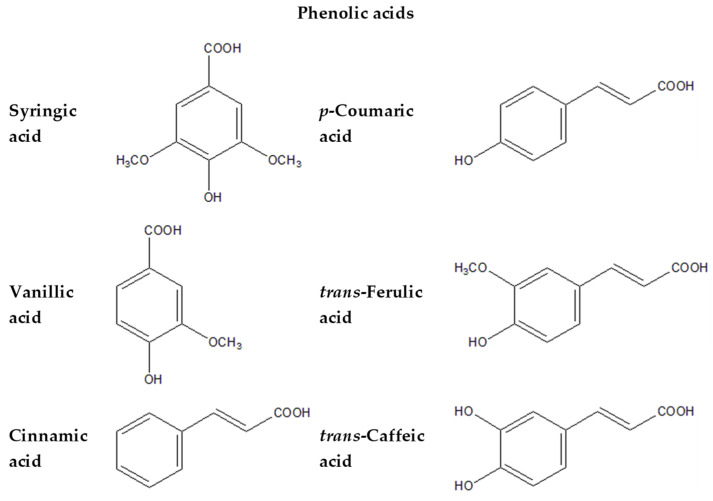
Structural formulas of primary phenolic compounds in *P. caerulea*.

**Figure 4:**
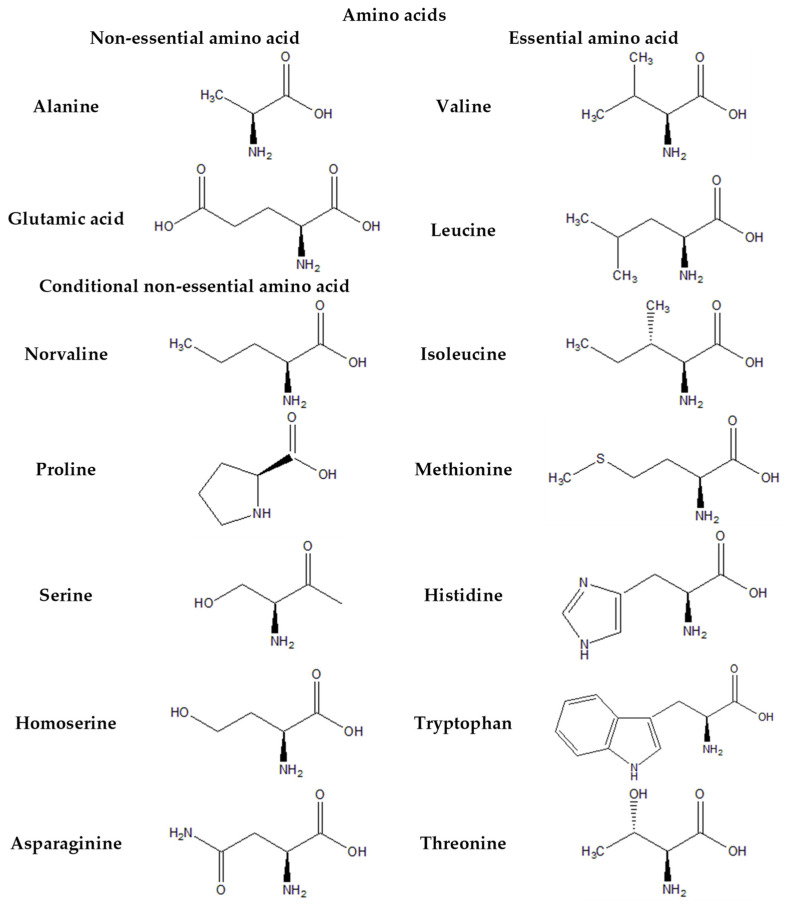
Structural formulas of essential amino acids in *Passiflora caerulea*.

**Figure 5:**
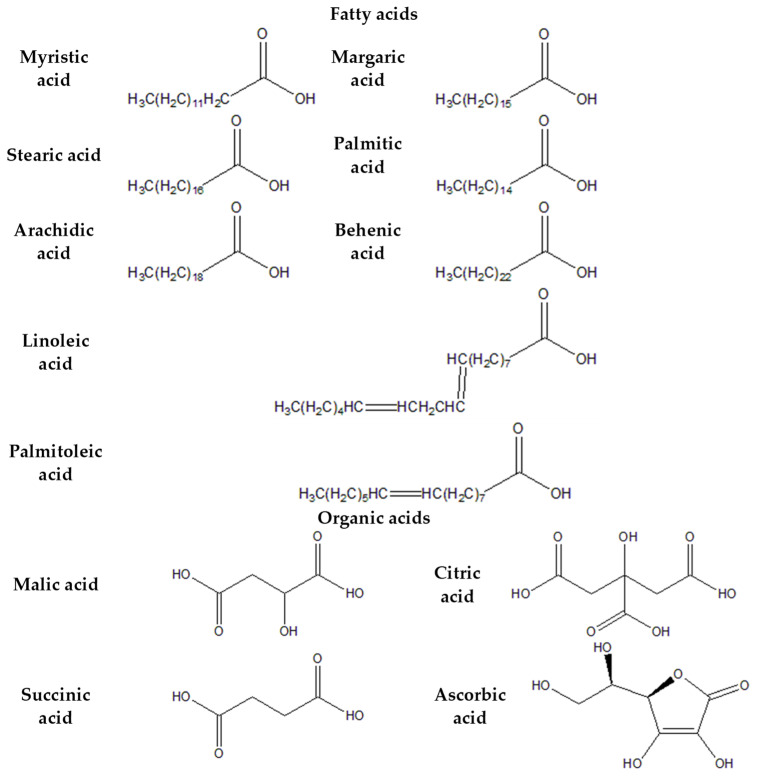
Structural formulas of essential organic and fatty acids in *P. caerulea*.

**Figure 6:**
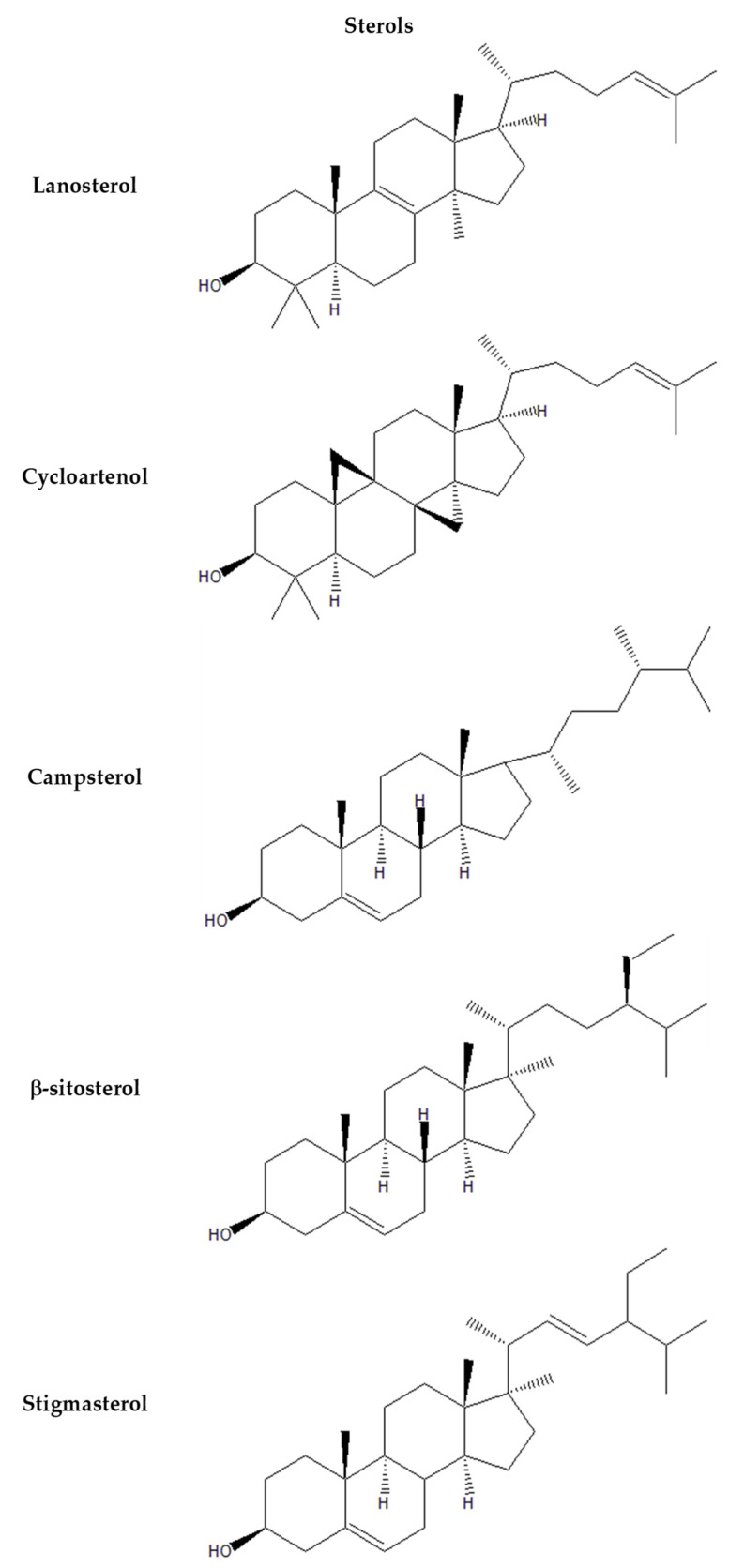
Structural formulas of major sterols in *P. caerulea*.

Figure 3, Figure 4, Figure 5 and Figure 6 provide the structural formulas of the compounds included in the composition of the leaves and pulp. 

## 4. Discussion

The content of three types of sugars in the leaves and pulp—sucrose, fructose and glucose—was determined (Table 1). Fructose predominated in pulp, followed by glucose. Sucrose was in minimal amounts, while maltose was absent. The results correlated with those reported by Song [41] but were different from those of De Oliveira [42], where sucrose was said to be the most common, followed by glucose and fructose. The three detected sugars are typical of passion fruit [43]. According to Shahbani et al. [44], glucose should be 18.00 g/100 g at the 55-to-60-day harvest stage to meet the standard of Brazilian legislation. The taste of passion fruit depends mainly on the individual sugars—glucose and fructose. SI and TSI were 24 and 16, while total sugar/TA was 30.10. This total sugar/TA ratio is responsible for the taste and aroma of the fruit and is an indicator of ripeness—commercial or sensory. These fruits can be defined as sweet according to this index. Carotenoids were found in traces in the fruits of *P. caerulea*. According to Wei et al. [45], the decrease in chlorophyll *a* content was the main reason for the decrease in chlorophyll content in *P. caerulea*. The accumulation of sugars mainly happened in the early stage of ripeness of fruit, and the dominant factors were fructose and glucose. 

In leaves, fructose dominated over glucose and sucrose (Table 1). Some authors found only glucose, deoxyhexose, pentose and dideoxyhexose in the leaves of the Polish representative of *P. caerulea* as part of glycosidic substitutes for flavone aglycones [16]. This is the first detailed study of the sugar composition and evaluation of SI and TSI of *P. caerulea* leaves and fruits. Leaves have lower SI and TSI than fruits due to lower sugar content. Their taste was more astringent and bitter, possibly due to their acid content and total phenolics. Chlorophylls were not found in fruits; they were only detected in leaves, which is related to photosynthesis.

The pH values for the pulp and leaves were 5.22 and 6.01 (Table 1). The titratable acidity in both samples was about 0.5. The data were comparable to the reported pH and titratable acidity of the mesocarp of *Passiflora quadrangularis* [44], but the pH values were higher than those reported for the fruits of *Passiflora cincinnati* Mas [46]. Ash content in pulp was about 6.25%, while in leaves, it was about 7.95%.

An extremely small number of articles discussing the elemental composition of *Passiflora* can be found in the literature. Song et al. determined the concentrations of nine elements in fresh fruits of *Passiflora foetida* in which potassium, phosphorus and magnesium had the highest concentrations (451 ± 7, 86 ± 7 и 40 ± 2 mg/100 g of FW) [41]. Other authors examine how the deficiency of various elements affects the content of Vitexin in the leaves of *Passiflora alata* Curtis [47] or how the low or high availability of various elements affects the development of the plant itself [48]. In relation to the evaluation of macro- and micro-minerals in crude drugs and infusions, the elemental composition of *P. caerulea* was determined and compared with four other herbs used as sedatives. [49]. In a review examining the current state of research related to *Passiflora* spp., the elemental composition of the pulp of seven different *Passiflora* species is included, one of which is *P. caerulea*. Results have been reported for the concentrations of a large number of elements in the pulp, including those determined in our study; for all elements, the results are consistent [50]. In the present work, the content of the elements was investigated not only in the pulp but also in the leaves. Except for copper, the remaining six elements have a lower concentration in the pulp than in the leaves, and the order of element concentrations changes from K > Mg > Na > Mn > Zn > Fe > Cu in the leaves to K > Mg > Na > Zn > Fe > Cu > Mn in the pulp.

The metabolic profile of the leaves and pulp of passionflower showed the presence of phenolic acids, amino acids, organic acids, fatty acids and phytosterol in the extracts. Four organic acids—malic, succinic, citric and ascorbic acids—were found in the leaves and pulp of *P. caerulea* (Table 2). The total content of organic acids in the pulp was about 1.9 times more than that in the leaves. The highest content in the leaves and pulp was ascorbic acid; it represented about 54% of the total content of organic acids in the leaves and 40% in the pulp. Citric acid was twice as much in the pulp as in the fruit. The pulp was also high in malic acid at 2.17 mg g^−1^ compared to <1 mg g^−1^ in the leaves. It was explained earlier that malic acid was the dominant factor in the later stage of color transformation when the acid degradation mainly occurred [45]. Ascorbic acid helps reduce free radicals and oxidative stress in cells. The combination of citric and ascorbic acid favors the strengthening of the immune system in humans. The presence of malic acid, which has the effect of a natural preservative, increases the stability of a food supplement when the pulp is included in it [51].

The presence of 12 amino acids was found in the pulp, and 9 were found in the leaves. Of the essential amino acids, the content of histidine is the highest (7.81 mg g^−1^) in the pulp. It is responsible for maintaining good muscle health and rapid recovery of the body after heavy physical exertion. The leaves have the highest tryptophan content (8.30 mg g^−1^). Therefore, the inclusion of the leaves in various nutritional supplement formulations could have a beneficial effect on sleep as well as the overall health of the body.

According to the results obtained (Table 3), it can be seen that in the fatty acids in the leaves and fruits of *P. caerulea*, saturated palmitic acid (C 16:0) predominates—17.1 mg g^−1^ and 13 mg g^−1^; a similar result was reported by Song at al. [41]. In terms of sterols, lanosterol predominates in the leaves at 10.1%, with β-sitosterol at 10.7% in the pulp. Lanosterol, β-sitosterol, stigmasterol and campesterol have anti-inflammatory properties and support the immune system to fight infectious and other diseases [52].

Due to their antimutagenic, antitumor and antioxidant properties, phenolic compounds are important for human health. The total phenolic content (TPC) varied in extracts from a given plant part using different solvents (Table 4). The total phenolic content (TPC) of passion fruit was usually above 1000 µg GAE g^−1^, making it a high-quality source of natural phenolic compounds [53]. For instance, the TPC content of the acetone extract from *P. leschenaultii* DC leaves collected in India was 440.24 mg GAE g^−1^ [54]. TPC for Brazilian native passion fruit *P. tenuifila* Killip pulp ranged from 9.21 to 13.25 mg GAE g^−1^ but was higher than Brazilian *P. edulis’s* TPC of 2.8 mg GAE g^−1^ [55]. Furthermore, Cao et al. [56] determined that the peel of Cambodian *P. edulis* x contained 32.6 ± 2.0 mg/g DW of TPC. Compared to blood orange extract (6 mg g^−1^) and lemon peel (7 mg g^−1^), it had a greater TPC content [57]. 

In our studies, the smallest TPC of *P. caerulea* was measured at 3.23 mg GAE g^−1^ for the methanolic extract of pulp, which is higher than that of *P. mollissima* (6.35 mg GAE/100 g) and *P. tarminiana* (10.18 mg GAE/100 g) [58]. The stated differences can be explained by different types of plant and environmental conditions [59,60]. The 70% ethanol extract showed the highest total phenolic content—13.68 ± 0.10 mg GAE g^−1^ dw—and the highest value of total flavonoid content—31.60 ± 0.25 μg QE g^−1^ dw. The phenolic content of the leaf extracts varied from 1.23 to 13.68 mg GAE g^−1^ dw. Another study showed that the number of phenolic compounds (expressed as mg GAE/g extract) was as follows: in *P. alata* (8.21 mg/g), *P. caerulea* (6.23 mg g^−1^) and *P. incarnata* (4.85 mg g^−1^) [61].

By all methods, the antioxidant activity of the leaf extracts was higher than that of the pulp. According to DPPH, FRAP and CUPRAC methods, extracts with 70% ethanol in the leaves have the highest values, while in the ABTS method, the value is the highest for 50%. In the pulp, the values are highest at 50% ethanol by all four methods.

Shanmugam et al. [62] evaluated the antioxidant activity by different assays and solvents of extract from *P. leschenaultii* DC leaves collected in India. The authors found that acetone showed the best recovery of bioactive compounds and antioxidant activity DPPH, ABTS (9760.44 ± 20.25 μM TE/g extract) and FRAP (1128.10 ± 7.30 mmol Fe (II)/mg extract). In our studies, the DPPH activity of the *P. caerulea* pulp (methanolic extract) exhibited the following data: 21.51 ± 0.27 mM TE g^−1^, ABTS 18.93 ± 0.60 mM TE g^−1^ and FRAP 7.56 ± 0.03 mM TE g^−1^.

*P. mollissima* or *P. tripartita* (Colombian banana passion fruit) extract showed antioxidant activity with IC_50_ of 3.8 ± 0.5 µg mL^−1^ and 50.1 ± 2.5 µg mL^−1^ based on DPPH and ABTS radicals assays, and 64.1 ± 3.4 µM Fe(II) g^−1^ based on the FRAP assay [63] 

Existing correlations between total phenolic and total flavonoid content and each of the considered AOA methods were checked using SPSS. The results are presented in Table 6. The highest linear correlation was observed between TPC and TFC with the DPPH method. Similar correlations have been observed by other authors for various medicinal plants [64].

Table 5 presents the results obtained from the studies of the antimicrobial activity of methanolic and ethanolic extracts of leaves and pulp of *P. caerulea*. In our studies, 50% methanolic and 70% ethanolic extracts of leaves and pulp of *P. caerulea* demonstrated limited antimicrobial activity compared to the controls, but the pulp extracts exhibited antifungal activity against *P. chrysogenum* and *F. moniliforme* ATCC 38932. Similar to our results for the antimicrobial activity of ethanolic extract of *Passiflora* species against *Bacillus subtilis,* ATCC 6633 was reported by Badalova et al. [65]. In other passionflower species, such as *P. quadrangularis, P. maliformis and P. edulis*, the methanolic extract did not show activity against Gram-negative bacteria [60]. A similar effect for *P. suberosa* was reported by Bandara et al. [66]. The antimicrobial activity of *P. caerulea* was investigated by Prithviraj et al. [67]. The authors stated that the methanolic leaf extract inhibited the growth of *B. cereus* (zone of 8 mm), while the methanolic leaf callus extract showed inhibitory activity against *E. coli* (zone of 8 mm), whose values were lower compared to our results. 

**Figure 7:**
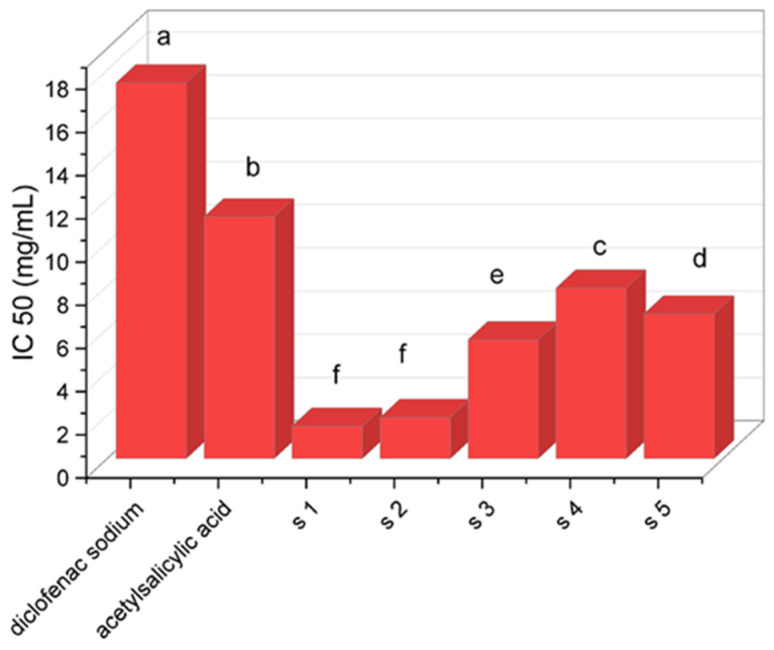
The inhibition of albumin denaturation activity of the *P. caerulea* extracts. Diclofenac and acetylsalicylic acid were used as standard. The results were expressed as IC_50_. s1—50% ethanol leaf extrac; s2—70% ethanol leaf extract; s3—methanol leaf extract; s4—50% ethanol pulp extract; s5—70% ethanol pulp extract.

In addition to antimicrobial potential, the extracts were investigated for in vitro anti-inflammatory activity for the first time using the prevention of protein denaturation. Protein denaturation is an indicator of the presence of an inflammatory process. During this process, the secondary and tertiary structures of the proteins are destroyed, and a violation of their biological functions is observed. In this regard, the ability of *P. caerulea* leaf and pulp extracts to prevent albumin denaturation was evaluated. Figure 7 presents the results as half maximal inhibitory concentration (IC_50_); The lower the value, the higher the anti-inflammatory activity. Nonsteroidal and steroidal anti-inflammatory drugs reduce inflammation, but these drugs have many unwanted side effects. Anti-inflammatory drugs are known to show a dose-dependent ability to inhibit thermally induced protein denaturation [68,69]. The results showed that the leaf extracts had better anti-inflammatory activity than the fruit extracts. The data also showed that all five samples (three leaves and two pulp extracts) had better albumin protection compared to two known anti-inflammatory drugs, diclofenac and acetylsalicylic acid. We found that 50% ethanol leaf extract (sample 1–1.5 mg mL^−1^) exhibited the highest anti-inflammatory activity, stronger than known anti-inflammatory drugs, followed by 70% ethanol leaf extract (sample 2–1.9 mg mL^−1^). Samples 3, 4 and 5 showed lower anti-inflammatory activity, but the methanol leaf extract (sample 3–5.5 mg mL^−1^) had better activity than sample 4 (50% ethanol pulp extract) and sample 5 (70% ethanol pulp extract). The methanol pulp extract did not show anti-inflammatory activity.

## 5. Conclusions

This study evaluated the metabolic profile, elemental composition, antioxidant, antimicrobial and anti-inflammatory activity of *Passiflora caerulea* L. growing in Bulgaria. The results showed that in the pulp and leaves of *P. caerulea*, glucose, fructose and sucrose were detected, and amino acids predominated in fruits. SI and TSI were evaluated as the main qualities of this fruit for human nutrition. Moreover, they have high potassium and magnesium values. The alcoholic extracts of the leaves showed higher values of AOA compared to the pulp, which is due to higher polyphenol and flavonoid content in them. As a result, the plant extracts also exhibited better anti-inflammatory and antimicrobial activity compared to those containing the pulp. All these results can serve as a good basis for the use of this plant in nutritional supplements, herbal infusions or the incorporation in food products.

## Figures and Tables

**Table 1:** Nutritional composition and plant pigments of the leaves and pulp of *P. caerulea*.

Characteristics	Leaves	Pulp
Moisture, %	8.64 ± 0.14	9.66 ± 0.10
Ash, %	7.95 ± 0.05	6.25 ± 0.06
pH	6.01 ± 0.02	5.22 ±0.03
Titratable acidity (TA), %	0.42 ± 0.05	0.47 ± 0.02
Glucose, %	1.51 ± 0.07	6.28 ± 0.05
Fructose, %	1.44 ± 0.20	7.50 ± 0.06
Sucrose, %	0.79 ± 0.19	0.37 ± 0.06
Fructose/glucose	0.95	1.19
Sweetness index (SI)	5.89	24.03
Total sweetness index (TSI)	4.10	16.39
Total sugar/TA	8.91	30.10
Chlorophyll *a*, µg g^−1^	412.3 ± 12.3	n.d *
Chlorophyll *b*, µg g^−1^	184.3 ± 18.9	n.d *
Total chlorophylls, µg g^−1^	596.6 ± 15.2	n.d *
Total carotenoids, µg g^−1^	7.9 ± 0.5	tr. *
Sodium, mg kg^−1^	1199 ± 56	225 ± 7
Potassium, mg kg^−1^	36,179 ± 515	23,946 ± 753
Iron, mg kg^−1^	81.0 ± 2.5	43.3 ± 3.0
Magnesium, mg kg^−1^	5064 ± 112	1890 ± 20
Manganese, mg kg^−1^	116 ± 3.0	5.15 ± 0.12
Copper, mg kg^−1^	7.29 ± 0.07	10.6 ± 0.6
Zinc, mg kg^−1^	88.8 ± 0.9	53.4 ± 2.1

* n.d.—not detected. tr—traces.

**Table 2:** Content of phenolic, organic and amino acids in *P. caerulea*.

	Leaf	Pulp
	Content ± SD, mg g^−1^	Content ± SD, mg g^−1^
Phenolic acids		
Syringic acid	3.31 ± 0.93	5.76 ±1.06
Vanillic acid	2.79 ± 0.78	6.84 ± 1.01
Cinnamic acid	0.55 ± 0.15	1.75 ± 0.26
*p*-Coumaric acid	1.47 ± 0.41	4.33 ± 0.64
*trans*-Ferulic acid	5.79 ± 1.64	9.86 ± 1.46
*trans*-Caffeic acid	0.50 ± 0.17	15.94 ± 1.73
Amino acids		
Non-essential amino acid		
L-Alanine	0.28 ± 0.09	1.31 ± 0.19
L-Glutamic acid	0.31 ± 0.09	1.48 ± 0.22
Essential amino acid		
L-Valine	0.35 ± 0.10	0.90 ± 0.13
L-Leucine	0.40 ± 0.11	1.63 ± 0.23
L-Isoleucine	0.32 ± 0.09	3.80 ± 0.56
L-Methionine	0.23 ± 0.06	nd
L-Histidine	nd	7.81 ± 1.24
L-Tryptophan	8.30 ± 1.46	3.25 ± 0.50
L-Threonine	0.46 ± 0.13	3.11 ± 0.46
Conditional non-essential amino acid		
Norvaline	nd	17.91 ± 3.29
Proline	nd	7.78 ± 1.15
L-Serine	0.62 ± 0.17	nd
L-Homoserine	nd	2.54 ± 0.37
L-Asparaginine	nd	3.92 ± 0.58
Organic acids		
Malic acid	0.6 ± 0.17	2.17 ± 0.32
Succinic acid	0.39 ± 0.11	0.94 ± 0.14
Citric acid	1.68 ± 0.47	3.48 ± 0.52
Ascorbic acid	3.09 ± 0.86	4.34 ± 0.64

**Table 3:** Content of fatty acids and sterols in the leaves and pulp of *P. caerulea*.

	Leaf	Pulp
	Content ± SD, mg g^−1^	Content ± SD, mg g^−1^
Fatty acids		
Miristic acid (C14:0)	2.21 ± 0.47	0.7 ± 0.15
Palmitoleic acid (C16:1)	0.4 ± 0.99	0.33 ± 0.07
Palmitic acid (C16:0)	17.09 ± 3.43	13.01 ± 2.75
Margaric acid (C17:0)	0.25 ± 0.05	0.18 ± 0.04
Linoleic acid (C18:2)	4.28 ± 0.92	5.98 ± 1.27
Stearic acid (C18:0)	3.73 ± 0.80	1.99 ± 0.42
Arachidic acid (C20:0)	1.87 ± 0.40	1.09 ± 0.23
Behenic acid (C22:0)	0.55 ± 0.12	0.82 ± 0.17
Sterols		
Lanosterol	10.23 ± 2.20	0.23 ± 0.06
Cycloartenol	1.50 ± 0.32	0.23 ± 0.05
Campesterol	0.40 ± 0.08	0.85 ± 0.18
β-Sitosterol	8.60 ± 1.88	10.42 ± 2.36
Stigmasterol	0.94 ± 0.20	0.20 ± 0.04

**Table 4:** Antioxidant activity, total phenolic content and total flavonoid content in extracts from leaves and pulp of *P. caerulea*.

Sample	TPC, mg GAE g^−1^	TFC, μg Qeb g^−1^	Antioxidant Activities, mM TE g^−1^			
			DPPH	ABTS	FRAP	CuPRAC
*P. caerulea* leaves (СН_3_ОН)	8.82 ± 0.03 ^c^	23.89 ± 2.85 ^b^	73.28 ± 2.12 ^c^	52.55 ± 0.58 ^c^	29.46 ± 2.00 ^b^	80.49 ± 7.36 ^b^
*P. caerulea* leaves (50% С_2_Н_5_ОН)	13.00 ± 0.03 ^b^	26.08 ± 2.45 ^b^	97.89 ± 5.45 ^b^	105.46 ± 3.60 ^a^	44.67 ± 2.50 ^a^	164.37 ± 8.28 ^a^
*P. caerulea* leaves (70% С_2_Н_5_ОН)	13.68 ± 0.10 ^a^	31.60 ± 0.25 ^a^	111.59 ± 2.77 ^a^	92.03 ± 0.29 ^b^	46.26 ± 1.75 ^a^	170.22 ± 7.36 ^a^
*P. caerulea* pulp (СН_3_ОН)	3.23 ± 0.08 ^f^	0.31 ± 0.01 ^c^	21.51 ± 0.27 ^e^	18.93 ± 0.60 ^f^	7.56 ± 0.03 ^d^	50.36 ± 0.10 ^d^
*P. caerulea* pulp (50% С_2_Н_5_ОН)	4.25 ± 0.05 ^d^	0.38 ± 0.01 ^c^	29.74 ± 0.57 ^d^	27.76 ± 0.80 ^d^	14.34 ± 0.06 ^c^	83.35 ± 0.09 ^b^
*P. caerulea* pulp (70% С_2_Н_5_ОН)	3.72 ± 0.02 ^e^	0.31 ± 0.01 ^c^	29.27 ± 0.27 ^d^	23.97 ± 0.57 ^e^	12.62 ± 0.04 ^c^	63.58 ± 0.06 ^c^

Means in a column with a common superscript letter (^a–f^) differ (*p* < 0.05) as analyzed by Duncan’s test.

**Table 5:** Antimicrobial activity of *P. caerulea* leaf and pulp extracts.

Test Microorganism	Inhibition Zones, mm									
	Leaf Extracts (10 mg mL^−1^)			Pulp Extracts (10 mg mL^−1^)			Controls * (10 mg mL^−1^)			
	СН_3_ОН	50% С_2_Н_5_ОН	70% С_2_Н_5_ОН	СН_3_ОН	50% С_2_Н_5_ОН	70% С_2_Н_5_ОН	A	P	N	F
*Bacillus subtilis* ATCC 6633	10.0 ± 0.00	10.5 ± 0.71	10.0 ± 0.00	8.0 ± 0.00	8.0 ± 0.00	8.0 ± 0.00	16.0 ± 0.00	-	n.a.	n.a.
*Bacillus cereus* NCTC 11145	10.0 ± 0.00	10.0 ± 0.00	9.0 ± 0.00	8.0 ± 0.00	8.0 ± 0.00	8.0 ± 0.00	20.0 ± 0.00	-	n.a.	n.a.
*Staphylococcus aureus* ATCC 25923	-	-	-	-	-	-	35.0 ± 0.00	30.0 ± 0.0	n.a.	n.a.
*Listeria monocytogenes* NBIMCC 8632	-	-	-	-	-	-	40.0 ± 0.00	12.0 ± 0.0	n.a.	n.a.
*Enterococcus faecalis* ATCC 29212	9.0 ± 0.00	10.0 ± 0.00	9.0 ± 0.00	-	-	-	38.0 ± 0.00	-	n.a.	n.a.
*Salmonella enteritidis* ATCC 13076	10.0 ± 0.00	9.5 ± 0.71	11.0 ± 0.00	9.0 ± 0.00	9.0 ± 0.00	8.0 ± 0.00	40.0 ± 0.00	-	n.a.	n.a.
*Klebsiella pneumoniae* ATCC 13883	-	-	-	-	-	-	25.0 ± 0.00	-	n.a.	n.a.
*Escherichia coli* ATCC 25922	10.0 ± 0.00	9.0 ± 0.00	9.0 ± 0.00	9.0 ± 0.00	9.0 ± 0.00	9.0 ± 0.00	16.0 ± 0.00	-	n.a.	n.a.
*Proteus vulgaris* ATCC 6380	-	-	-	-	-	-	30.0 ± 0.00	-	n.a.	n.a.
*Pseudomonas aeruginosa* ATCC 9027	10.0 ± 0.00	9.0 ± 0.00	9.0 ± 0.00	9.0 ± 0.00	9.0 ± 0.00	8.0 ± 0.00	16.0 ± 0.00	-	n.a.	n.a.
*Candida albicans* NBIMCC 74	-	-	-	-	-	-	n.a.	n.a.	22.0 ± 0.00	-
*Saccharomyces cerevisiae* ATCC 9763	-	-	-	-	-	-	n.a.	n.a.	31.0 ± 0.0	-
*Aspergillus niger* ATCC 1015	-	-	-	-	-	-	n.a.	n.a.	32.0 ± 0.0	25.0 ± 0.0
*Aspergillus flavus*	-	-	-	-	-	-	n.a.	n.a.	26.0 ± 0.0	20.0 ± 0.0
*Penicillium chrysogenum*	- -	-	-	11.0 ± 0.00	11.0 ± 0.00	10.0 ± 0.00	n.a.	n.a.	26.0 ± 0.0	13.0 ± 0.0
*Fusarium moniliforme* ATCC 38932	- -	-	-	9.0 ± 0.00	9.0 ± 0.00	9.0 ± 0.00	n.a.	n.a.	25.0 ± 0.0	-

* Controls: A—Ampicillin; P—Penicillin; N—Nystatin; F—Fluconazole.

**Table 6:** Correlation dependence between total phenolic and flavanol content and methods for determining AOA of different types of *P. caerulea* extracts.

	DPPH	ABTS	FRAP	CUPRAC
TPC	0.977	0.934	0.938	0.928
TFC	0.94	0.83	0.85	0.84

## Data Availability

Datasets from the time of this study are available from the respective authors upon reasonable request.
